# The readability of online health resources for phenylketonuria

**DOI:** 10.1007/s12687-020-00461-9

**Published:** 2020-03-27

**Authors:** Jessie M. Marsh, Thomas D. Dobbs, Hayley A. Hutchings

**Affiliations:** 1 Patient and Population Health and Informatics Research, Swansea University Medical School, Singleton Park, Swansea, SA2 8PP UK; 2 Reconstructive Surgery and Regenerative Medicine Research Group, Institute of Life Sciences, Swansea University Medical School, Swansea, SA2 8PP UK

**Keywords:** Online information, Readability, Phenylketonuria, PKU

## Abstract

Phenylketonuria (PKU) is a condition that results in the build-up of phenylalanine in the blood. This can cause severe brain damage and neurological issues if left untreated. Management can be complex and many individuals may turn to the internet to access further information. It is important that resources are understood as misinterpretation could result in harm to health. The aim of this study was to assess the readability of online resources for PKU and to assess their visual appearance using a communication sciences assessment framework. We searched the top five websites through Google using the search term “phenylketonuria/PKU”. We then analysed the text content of the identified websites using five readability formulae to determine the USA and UK reading grade. The median readability level across the five websites was US grade/UK grade 10.6/11.6, with individual grades ranging from 10/11 to 13.3/14.3. We found wide differences in the focus, layout and general appearance of the websites. The readability of resources was much higher than the recommended US 6th grade level. Online resources for PKU need to be simplified to ensure they can be easily understood.

## Introduction

Phenylketonuria (PKU) is one of the most common inborn errors of metabolism (Macleod and Ney 2010; Winn et al. 2016), with a mean European prevalence of around 1 in 10,000 newborns, although rates vary across Europe (Blau et al. 2010; van Wegberg et al. 2017). It is caused by an autosomal recessive deficiency in the phenylalanine hydroxylase (PAH) enzyme that is responsible for converting phenylalanine into tyrosine (van Wegberg et al. 2017). Deficiency of PAH leads to a build-up of phenylalanine in the blood and brain which is toxic and can lead to neurotransmitter dysfunction and motor defects (Blau et al. 2010; van Wegberg et al. 2017).

Left untreated PKU is associated with progressive intellectual impairment along with a number of additional symptoms including eczematous rash, autism, seizures, motor deficits and microcephaly (Blau et al. 2010; van Wegberg et al. 2017). As the child grows further developmental problems, aberrant behaviour and psychiatric symptoms become more apparent (Blau et al. 2010). In adults, PKU can present itself with a short attention span and low mood, confusion and, in extreme cases, seizures (Brown and Lichter-Konecki 2016).

If blood phenylalanine levels are controlled, the associated central nervous system defects can be effectively managed, with most individuals showing normal development and expected educational achievement (Blau et al. 2010). The management of PKU is dependent on maintaining a diet low in phenylalanine (Macleod and Ney 2010).

Recent studies however have illustrated that large number of patients have higher than recommended phenylalanine levels, especially with increasing age (Jurecki et al. 2017). They also have difficulty adhering to a low phenylalanine diet (Brown and Lichter-Konecki 2016).

Over the past two decades, there have been huge technological advancements in computing which have been accompanied by a rapid growth in the number of individuals having access to or using computers. There has similarly been an increase in the number and diversity of websites, with health information websites forming a large part. The increasing utilization of the internet has provided a better opportunity for people to search for health information online, where previously the major source of health information would be medical staff (Chen et al. 2018). It is estimated that 37% of all internet traffic relates to health information (Maloney et al. 2005). Many patients with PKU feel that they receive limited support from health professionals (Ford et al. 2018). This may result in PKU patients seeking additional guidance and information through online resources.

People with low literacy skills may not be able to read a book or newspaper, understand road signs or price labels, make sense of a bus or train timetable, fill out a form, read instructions on medicines or use the internet (The National Literacy Trust 2019). A high proportion of the adult general population has been documented as having below average levels of general literacy. Table 1 illustrates readability ages and the equivalent UK and US education level. Figures from the UK range from 1 in 8 to 1 in 4 adults having general literacy levels below that expected of a 6th grade student (age 11) (The National Literacy Trust 2019). In the USA, 52% of the population are documented as having only basic (4th or 5th grade) or below levels of literacy (Wylie Communications 2019).

**Table 1:** Readability ages and equivalent grades of education

Readability age	School grade	
	UK	US/Canada
0–2		
2–3	Nursery	
3–4	Nursery	
4–5	Reception	
5–6	1	Kindergarten
6–7	2	1
7–8	3	2
8–9	4	3
9–10	5	4
10–11	6	5
11–12	7	6
12–13	8	7
13–14	9	8
14–15	10	9
15–16	11	10
16–17	12	11
17–18	13	12
18+ University Degree or equivalent	13–16+	12–16+

Health literacy has been defined as ‘The degree to which individuals have the capacity to obtain, process, and understand basic health information and services needed to make appropriate health decisions’ (Ratzan and Parker 2000). Given the increase in internet use, it is essential that the information presented to patients is accessible and understandable to them. Poor reading ability has a major effect on the level of health literacy. As PKU may present with developmental and neurological problems in patients it is important that they receive accurate and understandable online information. Even subtle abnormalities in phenylalanine levels have been shown to impair ‘executive function’ such as planning, problem solving and information processing, making difficult to read information unavailable to them and potentially worsening their health inequality (Blau et al. 2010; VanZutphen et al. 2007). For patients who are experiencing some of the PKU symptoms this may make understanding information from websites even more difficult. The level of knowledge and information possessed by patients has also been shown to have an impact on treatment compliance. Accessibility of information is therefore key if patients are to make informed decisions (Fowler et al. 2011; Martin et al. 2005). The aim of this research was therefore to assess the readability of the major online resources for PKU and to assess their visual appearance.

## Methods

We undertook a scoping review of the literature prior to deciding which readability formulae to use. As we were assessing health related literature we aimed to determine the most appropriate formulae for this type of literature. In our selection of formulae we also considered the output and the ease of comparison of the readability assessment across different formulae. It has been demonstrated that for health-related literature where 100% comprehension is the goal, a combination of two or more formulae should be used, including the SMOG (Burke and Greenberg 2010; Wang et al. 2013). The five formulae chosen all display a calculated level of readability as a US grade level, enabling comparison across results. In addition, they could all be easily calculated using the freely available software, ‘readable’ (https://readable.com/). The readability formulae selected use a variety of techniques to calculate grades, some using whole text, while others use a sample of text (see Table 2).

**Table 2:** Readability formulae aims and equations

Formula	Author	Main aim of formula	
Flesch-Kincaid Grade level (Kincaid et al. 1975)	Rudolph Flesch	General readability calculation for everyday use	$0.39\ \left(\frac{\mathrm{total}\ \mathrm{words}}{\mathrm{total}\ \mathrm{sentences}}\right)$ + 11.8$\Big(\ \frac{\mathrm{total}\ \mathrm{syllables}}{\mathrm{total}\ \mathrm{words}}$) − 15.59
Gunning Fog Index (Gunning 1952)	Robert Gunning	Originally used to ensure newspapers and magazines were written to a suitable level	$0.4\ \Big[\left(\frac{\mathrm{words}}{\mathrm{sentences}}\right)$ + 100$\Big(\ \frac{\mathrm{complex}\ \mathrm{words}\ast }{\mathrm{words}}$)]
Coleman-Liau Index (Coleman and Liau 1975)	Meri Coleman and Ti L Liau	Used in the US office of education to help write school textbooks to suitable levels for specific ages	0.0588ANL – 0.296ANS – 15.8
Simplified measure of Gobbledygook (SMOG) (McLaughlin 1969)	G Harry McLaughlin	A quick, simple formula for general use	1.0430√( number of polysyllables × $\frac{30}{\mathrm{number}\ \mathrm{of}\ \mathrm{sentences}}\Big)+3.1291$
Automated Readability Index (Smith and Senter 1967)	EA Smith and RJ Senter	Made to ensure materials for the Air Force were more easily understandable—originally made as an attachment for type writers	$4.71\ \left(\frac{\mathrm{characters}}{\mathrm{words}}\right)$ + 0.5$\Big(\ \frac{\mathrm{words}}{\mathrm{sentences}}$) – 21.43

*ANL* average number of letters per 100 words, *ANS* average number of syllables per 100 word sample

*Complex words classified as words of 3 or more syllables

We performed a search using the terms ‘Phenylkentonuria’ and ‘PKU’ through Google. We wanted to identify the top five non-sponsored websites that were identified on the first search page, as data indicates that less than 10% of users progress beyond the first page of an internet search (https://www.protofuse.com/blog/details/first-page-of-google-by-the-numbers/). We focused on sites that had the primary purpose of providing information for patients about the condition. We excluded Wikipedia due to concerns about information accuracy and credibility (Gorman 2007; Luyt and Tan 2010). Google was chosen as it was deemed to be the most popular search engine at over 90% of the UK market share (Statcounter GlobalStats 2014). We undertook the searches using incognito to avoid any browser history bias. The searches and data capture were all undertaken on 10 October 2018 using the same computer.

We tabulated the top five identified websites into Excel (Microsoft Excel for Windows, Microsoft Office ProPlus 365). We then accessed each website one by one and pasted the text from the first or main information page (where multiple pages were present) of each website into a Word document (Microsoft Word for Windows, Microsoft Office ProPlus 365). We also took a screenshot from each website which was saved for later visual assessment.

We assessed the readability of the text from each of the websites with the Flesh-Kincaid Grade Level, The Gunning-Fog Index, The Coleman-Liau Index, The SMOG Index and the Automated Readability Index using an online calculator (https://readable.io). Readability formulae take different elements into account when calculating a score, therefore the text were edited prior to pasting into the readability calculator to avoid bias between the different formulae. Headings, subheadings, bullet points and hyphenated words from all website text were removed. Any identified spelling errors were also corrected. Once edited, we pasted the text from each of the websites into the calculator and calculated the five readability grades for each of the five websites. The calculations were repeated three times by two members of the team (JMM and HAH) to ensure that the results obtained were consistent.

### Data analysis

We analysed the readability data using SPSS (IBM SPSS Statistics for Windows, 2017. Version 25.0 licensed to Swansea University). The results are presented as individual readability grades for each of the five readability calculations for the five websites. We also present the pooled median readability grades with range for each of the websites identified for the purpose of comparison. The Kruskal-Wallis test was used to compare the median readability grades across each of the five top websites identified.

In addition to comparing the readability grades across websites, we also assessed the appearance of each of the websites using a framework based on guidance provided in the Centers for Medicare and Medicaid Services toolkit (https://www.cms.gov/Outreach-and-Education/Outreach/WrittenMaterialsToolkit/index) (Doak et al. 1996). Broadly, we assessed: overall design and page layout; fonts, size of print and contrast; headings, bulleted lists and blocks of text; use of colour; photographs and illustrations; and use of tables, charts and diagrams. We assigned a score of 1 where there was clear evidence of the criterion being met, − 1 where it was not met, and 0 where it was not applicable or unclear. We calculated an overall score for each website based on its visual appearance. We also documented the currency of the information and credibility by exploring website update information and whether source reference information were provided.

## Results

The top 5 websites identified using the search term ‘Phenylketonuria’ using Google incognito were, in order of appearance: The National Health Service (NHS); Genetics Home Reference (GHR); The National Society for Phenylketonuria (NSPKU); Healthline; and Mayo Clinic. When we used the abbreviation PKU, the same five websites were identified in the same order.

### Readability assessment

Table 3 illustrates the US and UK readability grades using five readability formulae for the top five sites identified. We found no difference in the results obtained when the process was repeated on three occasions and by different research team members. The median readability grade across the five websites using the five formulae was US 10.6 (UK 11.6). This was equivalent to a reading age of 15–16 years. The readability grade varied with different formulae, ranging between US 10 and 11.3 (UK 11 and 12.3).

**Table 3:** US and UK readability grades with equivalent age for the top five listed websites using the Flesch-Kincaid, Gunning Fog, Coleman-Liau, SMOG and Automated Readability Index

	GHR			Healthline			NHS			NSPKU			Mayo Clinic				
	Grade (USA)	Grade (UK)	Age (years)	Grade (USA)	Grade (UK)	Age (years)	Grade (USA)	Grade (UK)	Age (years)	Grade (USA)	Grade (UK)	Age (years)	Grade (USA)	Grade (UK)	Age (years)	Median grade (USA)	Median grade (UK)
Flesch-Kincaid Grade Level	12.4	13.4	17–18	8.2	9.2	13–14	9.2	10.2	14–15	10.1	11.1	15–16	9.2	10.2	14–15	9.82	10.82
Gunning Fog Index	13.9	14.9	University	10	11	15–16	11.5	12.5	16–17	12.1	13.1	17–18	10.6	11.6	15–16	11.62	12.62
Coleman-Liau Index	13.4	14.4	University	10.5	11.5	15–16	10.6	11.6	15–16	11.3	12.3	16–17	11.3	12.3	16–17	11.42	12.42
SMOG Index	13.3	14.3	University	10.6	11.6	15–16	11.5	12.5	16–17	12.7	13.7	17–18	11.3	12.3	16–17	11.88	12.88
Automated Readability Index	12.1	13.1	17–18	7.7	8.7	12–13	9.1	10.1	14–15	9.5	10.5	14–15	8.6	9.6	13–14	9.4	10.4
Median grade	13.3	14.3	University	10.0	11.0	15–16	10.6	11.6	15–16	11.3	12.3	17–18	10.6	11.6	16–17	10.83	11.83

In terms of the readability of each of the websites we identified that Healthline had the lowest readability grade, having a median readability grade of US 10 (UK 11) with a range between US 7.7 and 10.6 (UK 8.7 and 11.6). This was equivalent to a reading age of between 15 and 16 years. The most difficult website to read was GHR, with a median readability grade of US 13.3 (UK 14.4) and a range between US 12.1 and 13.9 (UK 13.1 and 14.9). This was equivalent to a University level reading age (18+).

Using the Kruskal-Wallis test, we identified that there was a statistically significant difference between the median readability grades across the five websites (*p* = 0.009). A post hoc analysis identified that the statistically significant difference in readability grades was between the GHR and Healthline websites (*p* = 0.006).

### Visual assessment

Table 4 illustrates the visual scoring assessment of each website based on its visual appearance. Of the five websites identified, NHS and Healthline appeared to be the most focussed on improving the individual’s health. The GHR website appeared to have a more specific focus on those searching for genetic information. The Mayo Clinic seemed at first to be targeted to both researchers as well as the public; however, on closer examination it was clear from the website that the text was more directed towards patients themselves. The aim of the NSPKU website was the management of PKU by patients and included lots of self-help and support information. The first pages of the identified websites which were used for the analysis of readability all included a background to PKU, PKU symptoms and how to manage the condition. The NHS website was the highest scoring in terms of its overall layout, followed by GHR and then Mayo Clinic. Healthline and NSPKU were jointly scored last in terms of overall layout.

**Table 4:** Visual assessment of top five listed websites (adapted from the CMS.gov toolkit for making material clear and effective) (US Department of Health and Human Services and Office of Disease Prevention and Promotion 2010))

Assessment criteria	National Health Service (NHS)	Genetics Home Reference (GHR)	The National Society for Phenylketonuria (NSPKU)	Healthline	Mayo Clinic
Overall design and page layout					
The size, shape and general look of the material was designed with its purpose and users in mind.	1	0	1	1	1
The material looks appealing at first glance.	0	1	1	1	1
A clear and obvious path has been created for the eye to follow.	1	1	1	1	1
The material that has a clear and consistent style and structure	1	1	1	1	1
Fonts, size of print and contrast					
For the regular text a font that is designed for ease of reading is used.	1	1	1	1	1
For headings, an easy-to-read font is used that contrasts with the main text	1	1	1	1	1
In general, no more than 2 or 3 different typefaces are used	1	1	1	1	1
The font size is large enough for the intended audience	1	1	1	1	1
Upper and lower case are used, not all capitals	1	1	1	1	1
To emphasize words and phrases italics or bold text are used	1	1	−1	1	1
For ease of reading, dark-coloured text is used on a very light background	1	1	1	1	1
Text is not aligned sideways, on patterned or shaded background or on top of photos or other images	1	1	1	1	1
For ease of reading, extra line spacing has been added	1	1	−1	1	1
For ease of reading, left justification is used throughout	1	1	−1	1	1
Lines of text are an appropriate length—neither too long or too short	1	1	1	1	1
Hyphenation has been avoided at the end of lines	1	1	1	1	1
Headings, bulleted lists and blocks of text					
There is a clear hierarchy of prominent headings and sub-headings	1	1	0	1	1
Contrast is used to make the main points stand out	1	1	1	1	1
Bulleted lists are well formatted	1	1	0	1	1
Effective ways are used to emphasize important blocks of text	1	1	0	0	1
Use of colour					
Colours used are appealing to the intended readers	1	1	1	1	1
Colour is used sparingly and in a consistent and deliberate way	1	1	1	1	1
The colour scheme works from a design standpoint and when printed	1	1	1	1	1
The colour scheme works for with diminished or limited colour perception.	1	1	0	−1	−1
Photographs and illustrations					
Photos and illustrations are used that relate directly to the information to reinforce key messages	0	−1	1	−1	1
Images used are clear, uncluttered and consistent in style	0	1	0	−1	−1
Photos and illustrations used are culturally appropriate for the intended readers	0	1	0	−1	1
When images include people, they are appropriate to the situation and intended audience	0	0	0	−1	0
Tables, charts and diagrams					
Likely literacy levels of the reader have been considered in the use of tables, charts and diagrams	0	−1	0	0	0
Titles, headings and other labelling is specific and clear	0	−1	0	0	0
A clean, uncluttered layout is used with strong visual cures to guide the reader through the information	0	−1	0	0	0
Numbers or calculations are carefully explained	0	−1	0	0	0
General information about website					
Last updated date given	1	1	− 1	− 1	− 1
Frequency of updates given	1	1	− 1	− 1	− 1
Relevant references given	− 1	1	1	− 1	1
Overall assessment score	24	23	14	14	22
Overall assessment summary	Well formatted in terms of font, style and layout. Clear from a language perspective with a focus on the patient. No photographs, illustrations, figures or tables to reinforce the message from the text. Text presented on one page with links to relevant other pages/topics	Clear layout in terms of colour, font size and style. Main section provides an overview with a series of sections appearing as headings which explode to provide information. Additional tabs that provide general genetic information. Language quite complex and technical. Figure with no explanation.	Summary information page was clear with good use of colour contrast. The language was straightforward with limited technical language used. The information was brief. On large photograph used which made the web page more accessible and appealing. Additional tabs included to provide reference information, support and recipes for example.	Clear layout with good use of fonts and headings. Language used was clear and straightforward. Information presented on one page with links to other clinical conditions highlighted and linked to other pages. No illustrations or figures but some photographs, but were related to other conditions or were advertisements.	Well presented with dark text on a light background. Some illustrations that were explained. Text presented on one page. Looks slightly cluttered in parts with advertisements to other Mayo Clinic services or requests to make a clinic appointment.

1 = evidence of criterion achieved; − 1 = evidence that criterion not achieved; 0 = no evidence, unclear or not applicable

#### NHS (https://www.nhs.uk/conditions/phenylketonuria/)

The NHS website provided information on different diseases and conditions and how to manage them. In terms of the general layout of the websites, the NHS was the most appealing and straightforward to read and to look at. The contrast was good and used a white background with clear bold headings in a black colour scheme. The text was well spaced out and the font size used was larger than the other sites. The language was clear and unambiguous. No images or diagrams were used however, which could have improved the overall visual aesthetics of the website. The information was up to date and included a regular review date. The information was contained within one page, with links out to relevant other pages. No references were included. The NHS site scored the highest in terms of its overall layout.

#### Genetics Home Reference (GHR) (https://ghr.nlm.nih.gov/condition/phenylketonuria)

GHR provided a guide for different genetic conditions, what they are and how to manage them. GHR looked professional and well laid out, with clear dark headings on a white background. The text however was complex and technical and included a ‘chemical structure’ diagram of phenylalanine as well as the phenylalanine hydroxylase (PAH) gene and a depiction of autosomal inheritance with no explanation offered. The information was contained under a series of headings that had to be clicked to provide detail. The website provided the date it was last updated and a review date. Source references for the information provided were also included. GHR scored highly in terms of overall layout.

#### The National Society for Phenylketonuria (NSPKU) (http://www.nspku.org/information/whatispku)

NSPKU is a charity dedicated to PKU individuals. The website included what PKU is and how to manage it, recipes and helpful advice for new parents with children with PKU. The NSPKU website had a dark green colour scheme for headings on a white background. The main information page was clear and the language was straightforward with limited technical language. A large photograph was used which made the web page more accessible and appealing. The page included additional tabs linking to reference information, support and recipes for example. NSPKU was one of the poorest scoring websites in terms of its overall appearance.

#### Healthline (https://www.healthline.com/health/phenylketonuria#symptoms)

The Healthline website contained general information and wellbeing tips. Information was presented on a simple white background with key headings. There were images present; however, none were relevant to PKU. The site contained lots of advertisements throughout the page including a large advertisement at the top of the page, obscuring sight of some of the website’s content. Healthline scored poorly in terms of its overall layout.

#### Mayo Clinic (https://www.mayoclinic.org/diseasesconditions/phenylketonuria/symptoms-causes/syc-20376302)

Mayo Clinic website was clear and well spaced out with text appearing on a white background. It included an image of a simple autosomal inheritance diagram that was explained further in the text. It also included some Mayo Clinic–related adverts and an opportunity to book a clinic appointment. Mayo Clinic scored well in terms of its overall layout.

## Discussion

Using the search terms ‘phenylketonuria/PKU’ through the Google incognito search engine, we identified that the top five websites for PKU were (in order): The National Health Service (NHS); Genetics Home Reference (GHR); The National Society for Phenylketonuria (NSPKU); Healthline; and Mayo Clinic. The median US readability grade across all five websites was 10.6, with individual grades ranging from 10 to 13.3. This was equivalent to a reading age of between 15 and 16 years. We identified a range of scores across sites with the Healthline website being identified as the simplest and GHR as the most complex. Visually, we deemed the NHS website to be the most appealing and simplest to read. This was based on a detailed visual assessment using an adapted framework that assessed various aspects of the written material including text size and colour, use of white space and inclusion of illustrations (US Department of Health and Human Services Centers for Medicare and Medicaid Services 2020). Based on these findings we would recommend that patients explore non-sponsored, governmental or charity websites as they had the best readability levels and visual assessments.

Our findings identified that the five websites all had readability grades above the recommended US 6th grade (age 11–12). Given the increasing extent that the internet is relied upon to further information and understanding of health conditions (Fox 2011), it is becoming increasingly important that such health resources are accessible to the general population. It is recommended that all patient information should be written at or below the US 6th grade level (US Department of Health and Human Services and Office of Disease Prevention and Promotion 2010). Given that between 1 in 4 and 1 in 6 adults in the UK have general health literacy levels below that expected of a 6th grade student (age 11) (The National Literacy Trust 2019), a substantial proportion of the UK population would struggle to read and understand the information contained in the identified websites for PKU.

Our findings concur with those from other studies that have demonstrated overly high reading levels of online resources for other health conditions, including skin cancer (Dobbs et al. 2017), breast cancer (Vargas et al. 2014a), breast reconstruction (Vargas et al. 2014c), abdominoplasty (Phillips et al. 2015), hernia repair (Vargas et al. 2014b), neurosurgery (Agarwal et al. 2013), stroke (Sharma et al. 2014), dementia (Weih et al. 2008), Parkinson’s disease (Fitzsimmons et al. 2010) and Polycystic Ovary Syndrome (Chiu et al. 2018). This highlights that high readability level is an issue across a range of health conditions.

Our findings are limited by a number of factors. We only used the search terms phenylketonuria or PKU. Including additional terms may have resulted in different results. We also only used five readability tools, using other tools may have resulted in different findings. We used the Google search engine and assessed the first or main page of each web site. Google is however the most frequently used in the UK (Statcounter GlobalStats 2014), and it is questionable whether other search engines would have changed the results. Including additional webpages may have changed the overall readability level, but we believe that most patients are unlikely to go on and look at further pages if they do not understand the first page. In addition, for many of the websites, the information was contained within one page.

Readability forms only one aspect of website assessment and is based primarily on information such as the sentence length, number of words per sentence and the complexity of some of the words. Other aspects of website also need to be considered such as the credibility or trustworthiness of the information as well as the quality of the information provided. We did not assess the quality, accuracy or relevance of the information published and this would need to be examined in a future study.

The readability formulae examine the readability of a piece of text. They do not include materials such as tables, diagrams, videos and audio-visual materials (Badarudeen and Sabharwal 2010), which may aid the understanding of the included text on websites. The readability grades found in our study therefore only give an insight into the overall website readability. We did however visually assess the overall appearance of each website which will also play a part in engaging with the patient.

Our findings for PKU websites, illustrate that there is a need to improve the readability of website information, particularly those that are regularly accessed by patients. There are a number of ways in which this can be achieved. The readability formulae are largely based on the number of words, sentence length and number of syllables per word. It may therefore be possible to reduce the readability grade by reducing these parameters (Dobbs et al. 2017). For example, by removing medical terminology (which is often complex and polysyllabic) from a piece of text the readability of it can be reduced (Sand-Jecklin 2007). For health information, however, this may not always be straightforward. It may not always be possible to remove medical terminology as an alternative may not exist, and not all medical terms are unfamiliar to patients. For example, a patient with PKU will understand the term.

Other ways of improving the visual appearance and accessibility of online materials are to consider the type and size of font (Doak et al. 1996; Eyles et al. 2003). The UK Government suggests that public resources should be written in Arial or Helvetica font (GOV.UK. Design System 2019). They also suggest using headings to break up text. Using bullet points and numbering, with large text and white space is also useful. Making new or complex words bold, along with a definition may also help readers (Danielson 1987).

Written text may also be improved by utilizing audio-visual materials. They can be an effective learning and teaching method and can convey and present information in a clear, organized way. In the health setting, several studies have shown benefits in using audio-visual materials alongside written materials (Carey et al. 2007).

When writing a piece of text, it is important to specify which aspects of it have a patient focus and which may encourage the patient to have further discussion with their clinician. Given the high proportion of individuals who are below normal levels of literacy, this should be aimed at 9–11-year-old reading capability.

## Conclusion

We found that the readability of online health resources for PKU appeared much higher than the recommended US 6th grade level. This high reading level may affect the management of PKU by patients. Online resources for PKU need to be re-worded into simpler, lower grade levels to ensure patients, caregivers and families can easily understand and interpret the text. Future studies should analyse other elements of readability, such as the use of audio-visual materials. The credibility and quality of the web content also needs to be considered.
